# Unraveling Molecular Mechanisms of THAP1 Missense Mutations in DYT6 Dystonia

**DOI:** 10.1007/s12031-020-01490-2

**Published:** 2020-02-28

**Authors:** Fubo Cheng, Michael Walter, Zinah Wassouf, Thomas Hentrich, Nicolas Casadei, Julia Schulze-Hentrich, Peter Barbuti, Rejko Krueger, Olaf Riess, Kathrin Grundmann-Hauser, Thomas Ott

**Affiliations:** 1 Institute of Medical Genetics and Applied Genomics, University Hospital of Tuebingen, Calwerstr. 7, 72076 Tuebingen, Germany; 2 Department of Neurology, The First Hospital of Jilin University, Changchun, People’s Republic of China; 3 Agilent Technologies, Stuttgart, Germany; 4 DFG NGS Competence Center Tuebingen (NCCT), University Hospital of Tuebingen, Tuebingen, Germany; 5 Luxembourg Centre for Systems Biomedicine (LCSB), University of Luxembourg, Belvaux, Luxembourg; 6 Parkinson Research Clinic, Centre Hospitalier de Luxembourg (CHL), Luxembourg Institute of Health (LIH), 1445 Strassen, Luxembourg

**Keywords:** DYT6 dystonia, THAP1, Missense mutation, Microarray analysis, Synaptic function, Protein stability

## Abstract

Mutations in *THAP1* (THAP domain-containing apoptosis-associated protein 1) are responsible for DYT6 dystonia. Until now, more than eighty different mutations in *THAP1* gene have been found in patients with primary dystonia, and two third of them are missense mutations. The potential pathogeneses of these missense mutations in human are largely elusive. In the present study, we generated stable transfected human neuronal cell lines expressing wild-type or mutated THAP1 proteins found in DYT6 patients. Transcriptional profiling using microarrays revealed a set of 28 common genes dysregulated in two mutated THAP1 (S21T and F81L) overexpression cell lines suggesting a common mechanism of these mutations. ChIP-seq showed that THAP1 can bind to the promoter of one of these genes, superoxide dismutase 2 (SOD2). Overexpression of THAP1 in SK-N-AS cells resulted in increased SOD2 protein expression, whereas fibroblasts from THAP1 patients have less SOD2 expression, which indicates that SOD2 is a direct target gene of THAP1. In addition, we show that some THAP1 mutations (C54Y and F81L) decrease the protein stability which might also be responsible for altered transcription regulation due to dosage insufficiency. Taking together, the current study showed different potential pathogenic mechanisms of *THAP1* mutations which lead to the same consequence of DYT6 dystonia.

**Electronic supplementary material:**

The online version of this article (10.1007/s12031-020-01490-2) contains supplementary material, which is available to authorized users.

## Introduction

Dystonias are a heterogeneous group of hyperkinetic movement disorders characterized by involuntary muscle contractions which result in twisting, repetitive movements, and abnormal postures (Fahn et al. 1987). It is the third most common movement disorder worldwide after Parkinson’s disease and essential tremor (Fahn et al. 1987). DYT6 is the second distinct form of primary dystonia after DYT1 dystonia. It has been initially referred to as dystonia of mixed type and has first been discovered in Amish-Mennonite families in 1997 (Saunders-Pullman et al. 2007). The disease locus, DYT6, has originally been assigned to 8p21-q22 by linkage analysis (Almasy et al. 1997) and subsequently to 8p11.21. In 2009, two distinct THAP1 mutations (p.F45fs73X and p.F81L) have been identified in five DYT6 dystonia families implicating this gene in DYT6 dystonia (Fuchs et al. 2009). The *THAP1* gene mutation has been screened for and found in primary dystonia patients of different populations (Bressman et al. 2009; Cheng et al. 2011). Until now, more than eighty different mutations have been found in *THAP1* gene in patients with primary dystonia. Around two third of them are missense mutations, but the potential pathogeneses of each mutations are largely elusive (see review LeDoux et al. 2012; Lohmann and Klein 2017).

The THAP1 protein belongs to the family of zinc finger-containing transcription factor and functions as a transcription factor (Roussigne et al. 2003; Bessière et al. 2008). Recently, comprehensive transcriptome analysis of mouse model for DYT6 dystonia showed altered expression of genes involved in many different pathways (Yellajoshyula et al. 2017; Zakirova et al. 2018; Frederick et al. 2019). However, the transcriptional functions of THAP1 in human neuronal cells are still unclear.

Here, we used microarray expression profiling and protein stability test to characterize potential molecular pathogenesis of different *THAP1* missense mutations found in DYT6 patients.

## Materials and Methods

### Cell Culture and Transfection

HEK-293 and SK-N-AS cells were grown in DMEM medium with 10% FBS and 1% Glutmax at 37 °C in a humidified atmosphere containing 5% CO2. Effectene transfection reagent (QIAGEN, Hilden, Germany) was used for transfection according to the instruction of manufacturer. For stably transfection, expression vector constructs (linearized by ScaI restriction enzyme) were stably transfected into SK-N-AS cells. Positive clones were selected using geneticin (G418) at a final concentration of 250 μg/ml. Expression levels of THAP1 in different clones were tested by western blot.

Human fibroblasts were collected from patients with *THAP1* mutation. Informed consent was obtained from all patients involved in our study prior to cell donation. Dermal fibroblasts, obtained from skin biopsies of three patients with *THAP1* mutation, were cultured as previously reported (Reinhardt et al. 2013). Control fibroblasts from three age and sex matched healthy controls were obtained from Biobank, Hertie Institute for Clinical Brain Research, Tuebingen, Germany. Using of human fibroblasts human samples was approved by ethic committee of University Hospital of Tuebingen.

### THAP1 Expression Vectors

The *THAP1* cDNA expression plasmids were generated as described previously (Cheng et al. 2014). The THAP1 mutations were introduced using in vitro mutagenesis methods using specific primers as described previously (Cheng et al. 2014). In current study, untagged wild-type and mutant (S21T, C54Y, F81L, L180S, and Q154fs180X) THAP1 expression vectors were generated using pcDNA3.1 empty vector. THAP1-flag vector was generated using pcDNA3.1/flag empty vector.

### Protein Extraction and Western Blotting

Total protein was extracted using RIPA buffer, while nuclear fraction was extracted using Nuclear Extraction Kit (Sigma) according to manufacturer’s instruction. Protein concentration was measured using BCA Protein Assays (Thermo Fisher Scientific). Western blot was performed as previously described (Cheng et al. 2014). Proteins were separated in SDS-PAGE gels (Bio-Rad) and transferred to nitrocellulose membranes. After incubated with primary antibody and secondary antibody, the blot membrane was directly detected in Odyssey® Fc Dual-Mode Imaging System. All western blots were repeated at least three times.

### RNA Isolation and Microarray Analysis

Total RNA of SK-N-AS cells were isolated using RNeasy Mini Kit (QIAGEN). Quality of RNA specimen was checked on an Agilent Bioanalyzer 2100 and processed for Affymetrix GeneChip® microarray hybridization using the GeneChip HT 3’ IVT Express Kit (Affymetrix) according to the manufacturer’s instructions. Fifteen microgram of labeled and fragmented cRNA was hybridized onto Affymetrix U219 GeneChip® array plates. Hybridization, staining, washing, and scanning were performed fully automated in an Affymetrix Gene Titan instrument. For each cell line/construct, three independent clones were analyzed. Additionally, a cell line transfected with the empty vector (three independent clones) was included in the experiment to determine the baseline expression level of all transcripts.

After visually inspecting scanned images for hybridization artifacts and proper grid alignment, AGCC 3.0 (Affymetrix) processed results were stored in CEL files. Further data analysis steps were carried out with in R (v2.14.09) using Bioconductor (v2.14.0). First, the complete expression information from every chip was background-corrected, normalized, and summarized with robust multichip average. The Bioconductor package *limma* was used to devise a linear model for gene expression as a function of influencing factors. Empirical Bayes shrinkage of standard errors was employed to derive the moderated F-statistic. The resulting *p* values underwent multiple testing corrections according to Benjamini-Hochberg. A decision matrix was produced through the function *decide tests* of the *limma* package to determine attribute significant changes in individual contrasts (|Log2| > 0.8; *p* < 0.005). Tables of differentially regulated transcripts were uploaded in Ingenuity Pathway Analysis (IPA, Ingenuity, Redwood City, CA) and analyzed for interactions and regulated genes against its internal databases.

### Total RNA Extraction and Reverse Transcription and Quantitative Real-Time PCR

Total RNA was extracted from SK-N-AS cells using the RNeasy Mini Kit (QIAGEN) according to the manufacturer’s instructions. After measuring the concentration on a NanoDrop 1000 Spectrophotometer (Thermo Scientific), RNA was stored at − 80 °C. Reverse transcription of mRNA into cDNA was performed by using Omniscript RT Kit (QIAGEN) according to the manufacturer’s instructions. A total of 500-ng RNA was used as template. The reaction mixture was incubated at 37 °C for 1 h. Synthesized cDNA was stored at − 20 °C before used for further analysis.

Quantitative real-time PCR (qPCR) was performed using the QuantiTect SYBR® Green PCR Kits (QIAGEN) according to the manufacturer’s instructions as described previously (Cheng et al. 2012a). In the analysis of the RNA expression, GAPDH and Actin were used as reference genes. Student’s *t* tests were used to compare RNA expression between experiment samples and control samples.

### Chromatin Immunoprecipitation and High-Throughput Sequencing (ChIP-Seq)

ChIP assays were performed according to previously published paper (Landt et al. 2012). Briefly, SK-N-AS cells stably overexpressing THAP1-flag protein were cross-linked to DNA by adding formaldehyde to a 1% final concentration. After sonicating and centrifuging, chromatin was incubated with rabbit anti-flag antibody (cell signaling) pre-incubated Dynabeads™ M-280 Sheep Anti-Rabbit IgG (Thermo Fisher Scientific). Anti-rabbit IgG was used as negative control. After washing steps, the precipitated immuno-complexes were treated with proteinase K, and DNA was purified by column. ChIP DNA was purified to generate ChIP-seq libraries using NEBNext® Ultra™ II DNA Library Prep Kit (New England Biolabs). Next-generation sequencing was done on Illumina NextSeq500 system using the 75 bp high output sequencing kit. ChIP-seq data analysis was performed in Galaxy platform (https://usegalaxy.org/). Briefly, ChIP-seq raw reads were aligned to the hg19 genome assembly using Bowtie2 (Langmead and Salzberg 2012) with the default parameters. Only tags that uniquely mapped to the genome were used for further analysis. ChIP-seq peaks were identified using MACS (Zhang et al. 2008).

### Luciferase Reporter Gene Assay

Luciferase reporter gene assays were performed as previously described (Cheng et al. 2014). The SOD2 promoter region was predicted using the Promoter2.0 and Web Promoter Scan Service software packages. The full-length SOD2 promoter region (driving expression of isoform: NM_001322819.2 and NM_001322820.2) was predicted by software (Promoter2.0 and Web Promoter Scan Service software packages) and PCR-amplified using specific primers from genomic DNA and inserted into the pGL3.0 reporter gene plasmid (Promega). Activities of firefly and Renilla luciferase were measured after 48 h of incubation with the Dual Luciferase Reporter Assay System (Promega) in a Mithras luminometer (Berthold Technologies). All experiments were verified in at least of three independent replicates.

### Assessment of Protein Stability

THAP1 protein stability was tested using a doxycycline dependent Tet-on system as previously reported (Vulinovic et al. 2014). SK-N-AS Tet-on cells (stably overexpression of tetracycline transactivator tTA protein) were established. After transfection of different pcDNA5/TO THAP1 (wild-type or mutants) vectors into SK-N-AS Tet-on cells, positive clones were selected using culture medium supplemented with blasticidin (7.5 μg/ml) and hygromycin (200 μg/ml). After detection of THAP1 expression level followed by adding doxycycline (1 μg/ml), different positive clones with the same expression level of THAP1 proteins (wild-type or mutants) were selected and used for protein stability assessment. For protein stability test, cells were incubated with culture medium with doxycycline (1 μg/ml) for 24 h, and fresh medium without doxycycline was replaced after washing with PBS twice. Cells were harvested at 0, 24, 48, and 72 h to test the level of different THAP1 proteins. All the experiments were performed at least three times independently.

## Results

### Confirmation of THAP1 Band and Subcellular Distribution

**Fig. 1:**
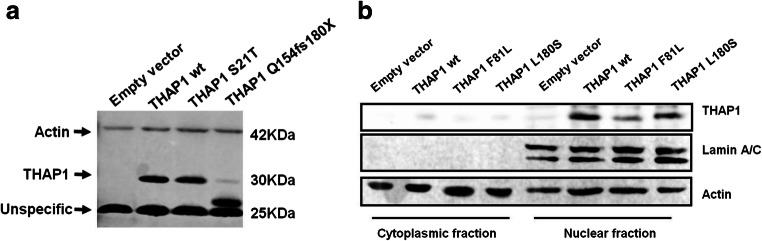
THAP1 molecular weight and subcellular distribution of THAP1. **a** Western blot showing a 30-kDa band and a 25-kDa band in HEK cells overexpressing wild-type THAP1 or F21L mutant THAP1 using the Proteintech antibody (12584-1-AP). A small band (around 27 kDa) was observed in HEK cells overexpressing Q154fs180X mutant THAP1. Mass spectrometry analysis detected two THAP1 peptides in the 30-kDa band (peptide 1, IHQLEQQVEK; peptide 2, LKEVVHFQK) but not in the 25-kDa band. **b** Wild-type and missense mutant (F81L and L180S) THAP1 protein distributed mainly in the nuclear fraction. Lamin A/C was used as marker of nuclear protein

Because there is no specific antibody for THAP1 available, we used HEK cells transiently overexpressing of THAP1 and mass spectrometry analysis (performed in the Quantitative Proteomics & Proteome Center Tuebingen) to test the specificity of currently used antibody in the literature (Gavarini et al. 2010). Using the THAP1 antibody from Proteintech (12584-1-AP), we found two bands (25 kDa and 30 kDa) in cells overexpressing wild-type or mutant THAP1 proteins (Fig. 1a). In cells overexpressing truncated THAP1 (Q154fs180X), we found a 25-kDa and a 27-kDa bands. This 27-kDa band could be the truncated THAP1 protein compared to bands found in wild-type THAP1 overexpressing cells (Fig. 1a). Mass spectrometry detected two THAP1 peptides in the 30 kDa band (peptide 1, IHQLEQQVEK; peptide 2, LKEVVHFQK). No THAP1 peptide was detected in the 25-kDa band. These results suggest the 30-kDa band to be the true wild-type THAP1 protein, while the 27 kDa band is truncated THAP1 protein product, while the 25 kDa band is an unspecific band. After separating the cytoplasmic and nuclear fraction, we observed that THAP1 proteins (both wild-type and several missense mutants) were predominantly found in the nuclear fraction (Fig. 1b).

### Transcriptome Analysis of Cell Models for DYT6 Dystonia

In order to assess the transcriptional dysregulation caused by *THAP1* mutations, we examined the effect of overexpression of four cloned vectors (empty vector, THAP1 wild-type, THAP1 S21T, and THAP1 F81L) on gene expression of stably transfected SK-N-AS cells. Gene expression levels were normalized to the empty vector group. Both S21T and F81L mutations were found in patients with primary dystonia and were also proved as pathogenic mutations (Fuchs et al. 2009; Gavarini et al. 2010).

Microarray array analysis showed 274 genes were significantly dysregulated in wild-type THAP1 overexpressing cell lines compared to empty vector transfected control lines (Table 1 and Supplementary microarray data), including 175 up- and 99 downregulated genes (Table 1). Subsequently, we compared the expression profiles of cell lines overexpressing mutant THAP1 (S21T and F81L) protein and wild-type THAP1 protein. We found that in S21T THAP1 overexpression cell lines, 272 genes were dysregulated, while only 73 genes were dysregulated in F81L THAP1 overexpression cells (Table 1).

**Table 1:** The number of significantly dysregulated genes compared with two different groups

Group comparison *	Number of genes up-regulated	Number of genes down-regulated
wild-type vs. Empty vector	175	99
S21T vs. wild-type	102	170
F81L vs. wild-type	24	49
Wild-type vs. mutant**	12	35

*wild-type: wild-type THAP1 overexpression cell line

**mutant: both S21T and F81L mutant THAP1 overexpression cell lines

Unexpectedly, we did not observe any expression change of two previously reported THAP1 targets (*TOR1A* (Gavarini et al. 2010; Kaiser et al. 2010) and *RRM1* (Cayrol et al. 2007)) when comparing the wild-type cell line with the empty vector cell line or comparing mutant cell lines with the wild-type cell line.

### Functional Network Analysis

Ingenuity Pathways Analysis (IPA) was used to classify genes according to their function and to reveal their associated cellular functions and networks. From the 274 significantly affected genes identified comparing the wild-type THAP1 cell line with the empty vector cell line, 266 genes mapped with the IPA database and revealed 17 significantly enriched networks (*p*adj < = 10E-7) (Supplementary Table 1). In the molecular function and cellular compartment analysis, “cellular growth and proliferation and embryonic development” showed the lowest *p* value and presented the most significantly important functional changes, which indicate the most important functions of overexpressed wild-type THAP1 protein in neuronal cells (Supplementary Fig. 1).

**Fig. 2:**
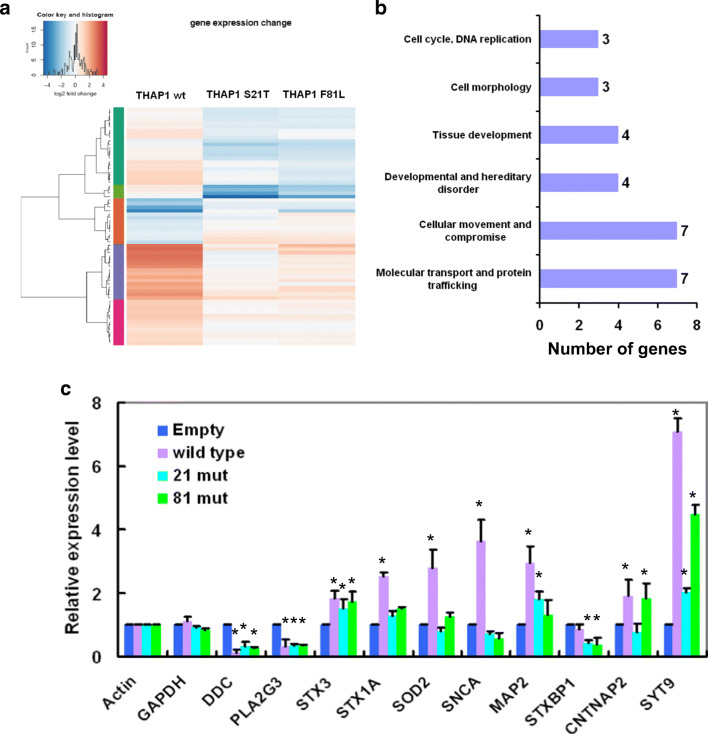
Transcriptome analysis of cell models for DYT6 dystonia. **a** Heatmap showing the expression values of genes differentially expressed between wild-type THAP1 and mutant THAP1 cell lines. **b** Networks involved in 28 overlap genes selected using a threshold of two-fold increase or decrease, six functional networks were identified. **c** Ten genes were selected from microarray data based on their function and confirmed by qPCR in different cell lines. (empty/empty vector overexpression cell line; wild-type/wild-type THAP1 overexpression cell line; 21 mut/THAP1 S21T mutant overexpression cell line; 81 mut/THAP1 F81L mutant overexpression cell line) (*p < 0.001 by comparing to empty vector overexpression cell line)

Regarding the effects of THAP1 mutations, the two mutant cell lines were compared with the wild-type THAP1 cell line to identify the gene expression changes caused by mutant proteins. A total of 47 dysregulated genes were found in common between both mutants compared to the wild-type THAP1 cell line, including 12 up- and 35 downregulated genes (Fig. 2a and Supplementary Table 2). These genes showed the same direction of change in both mutant cell lines. Using a threshold of > = 2-fold increase or decrease, a total of 28 genes were selected from this overlap, containing 21 down- and 7 upregulated genes (Supplementary Table 2). These overlapping genes indicate the common transcriptional dysregulation effects of the studied *THAP*1 mutations. Analyzing these genes in IPA revealed six networks (Fig. 2b and Supplementary Table 2). We observed the “molecular transport and protein trafficking” network to be the most significantly enriched molecular functions and cellular compartments, recruiting 7 genes in total (Fig. 2b and Supplementary Fig. 2). Some genes related to synaptic function, like *STXBP1*, *SNCA*, and *SOD2*, were also observed.

### Microarray Data Confirmation

Microarray data were validated by real-time qPCR for a subset of 10 genes using total RNA isolated from each cell line (Fig. 2c). Three genes related to neurological function (*STXBP1*, *SNCA*, and *SOD2*) by comparing wild-type THAP1 cell line with both mutant cell lines and seven candidates from dysregulated genes by overexpressing wild-type THAP1 based on gene function (related to neurological function or neurological disease) (*DDC*, *PLA2G3*, *STX3*, *STX1A*, *MAP2*, *CNTNAP2*, and*SYT9*) were selected to confirm the results of microarray. The real-time PCR results showed similar expression changes in gene expression to microarray analysis (Fig. 2c).

### THAP1 Directly Regulates SOD2 Expression

**Fig. 3:**
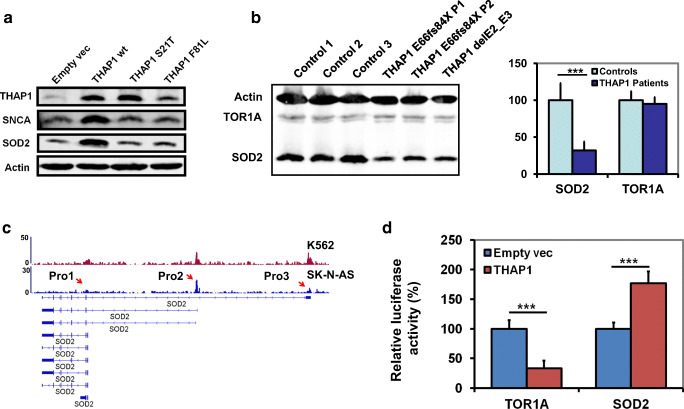
THAP1 directly regulates the expression of SOD2. **a** Western blot analysis showed that overexpression of wild-type THAP1 but not mutant THAP1 or empty vector leads to increased expression of SNCA and SOD2. **b** Decreased expression of SOD2 was observed in fibroblasts from DYT6 patients, and TOR1A protein level remains normal. **c** ChIP-seq showed THAP1 binds to *SOD2* promoter regions of different isoforms. THAP1 ChIP-seq of K562 was obtained from ENCODE database. (Pro1: promoter region of isoform 1). **d** Luciferase reporter analysis revealed overexpression THAP1 represses the activity of *TOR1A* promoter while upregulate the activity of *SOD2* promoter (Pro2). (*** *p* < 0.001)

Using microarray analysis, we observed the dysregulation of genes related to synaptic function after overexpression of THAP1, but it is unclear whether THAP1 directly regulate expression of these genes. First, we confirmed the protein level of two targets related to synaptic function (SNCA and SOD2) in different cell lines and found the changes were similar to the mRNA expression changes (Fig. 3a). THAP1 ChIP-seq database (ENCODE database) showed that THAP1 can bind to promoter regions of different SOD2 isoforms in K562 cells but not on SNCA promoter (Fig. 3c). In order to validate these results in human neuronal cells, we performed flag ChIP-seq in SK-N-AS cells overexpressing of flag-tagged THAP1 and found similar results (Fig. 3c). Furthermore, we performed luciferase reporter assay and found that overexpression of THAP1 can repress the *TOR1A* promoter activity as reported before but activate the activity of *SOD2* promoter (Pro2, promoter regions of NM_001322819.2 and NM_001322820.2) in SK-N-AS cells (Fig. 3d). In order to validate these results in human patient samples, we collect fibroblasts from three patients and three controls. Western blot results showed that the SOD2 but not TOR1A is significantly decreased in fibroblasts from THAP1 patients compared to normal controls (Fig. 3b).

### Protein Stability Assessment

**Fig. 4:**
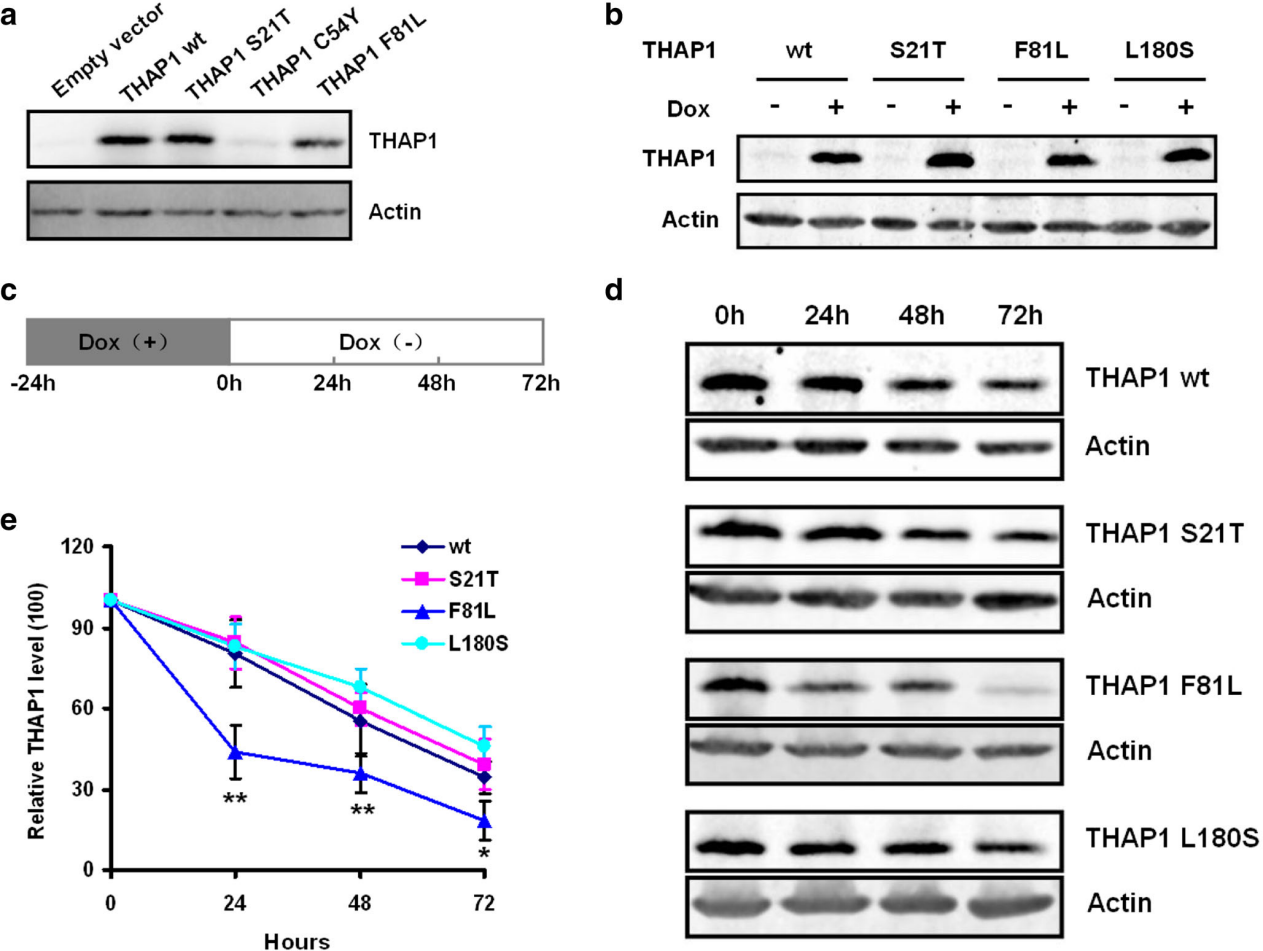
THAP1 Protein stability assessment. **a** The expression level of different mutated THAP1 proteins in transient transfected HEK cells. Wild-type and S21T THAP1 protein showed the similar expression level, the F81L THAP1 protein is lower than wild-type or S21T mutant protein, while almost no THAP1 mutant protein was detected in cells overexpression THAP1 C54Y construct. All cells were treated in the same conditions except different constructs were used for transfection. **b** SK-N-AS cell lines expression of the same level of different THAP1 proteins (wild-type or mutant) were selected after induced by adding doxycycline (Dox). **c** Diagram showed the strategy to test THAP1 protein stability. **d** and **e** THAP1 F81L protein degraded faster compared to wild-type or other mutated (S21T and L180S) THAP1 proteins at 24, 48, and 72 h. (*p < 0.01; **p < 0.001 by *t* test)

After transiently transfection of wild-type or mutant *THAP1*s in HEK cells, we observed lower THAP1 protein levels in cells overexpression of THAP1 C54Y or F81L mutant protein compared to cells overexpression wild-type or S21T mutant protein, while the C54Y protein level is even lower than F81L (Fig. 4a). These data might indicate decreased protein stability of C54Y and F81L mutant THAP1 protein. In order to prove this hypothesis, we performed protein stability assay using doxycycline inducible Tet-on system in SK-N-AS cell line. We found the THAP1 F81L mutant protein degraded faster than the wild-type, S21T, or L180S mutant proteins, which indicates reduced protein stability of F81L mutant THAP1 protein (Fig. 4b–e). No difference in protein stability was observed when comparing THAP1 wild-type protein to S21T or L180S mutant protein (Fig. 4e).

## Discussion

In the present study, we characterized the potential molecular pathogenesis of different *THAP1* missense mutations. Using microarray analysis, we observed that *THAP1* missense mutations cause dysregulation of genes involved in network of synaptic functions and THAP1 could directly regulate these genes. Protein stability analysis revealed decreased THAP1 protein stability with some missense mutations which leads to dysregulation of target genes due to protein dosage insufficiency. These results suggest that the consequence of all *THAP1* mutations are primary dystonia but the molecular pathogeneses of different *THAP1* missense mutations were different.

Alteration in protein intracellular localization was found to be one of the potential pathogenesis of *THAP1* mutations by us and others (Osmanovic et al. 2011; Cheng et al. 2012b). However, in present study, we did not observe drastic changes in protein intracellular localization in THAP1 protein with missense mutations affecting THAP domain (F81L) or coiled-coil domain (L180S) (Fig. 1b). This indicates that altered protein subcellular distribution might be restricted to truncated THAP1 mutant proteins affecting the nuclear localization signal (NLS) domain.

As a transcription factor, THAP1 can regulate expression of genes, while mutant THAP1 could lose these function and leads to dysregulation of target genes. Many dysregulated genes were found in cells or mouse brain tissues with THAP1 mutations or deletions. In HUVECs, pRB/E2F cell-cycle target genes like RRM1 were found dysregulated after THAP1 knockdown (Cayrol et al. 2007). Mouse THAP1 is essential for regulating genes related to embryonic stem cells (mESC) potential, survival, and proliferation (Aguilo et al. 2017). In mouse brain, *THAP1* mutations or deletions lead to dysregulation of genes involved in pathways of eIF2α signaling, mitochondrial dysfunction, and neuron projection development (Zakirova et al. 2018). Another study found that THAP1 is required for the timing of myelination initiation in oligodendrocyte during CNS maturation in mouse (Yellajoshyula et al. 2017). More recently, Frederick et al. (2019) have also analyzed the phenotypical consequences of a heterozygous loss-of-function mutation on transcriptional profiles. These mice show alterations in the expression of genes involved in nervous system development, synaptic transmission, cytoskeleton, gliosis, and dopamine signaling. However, to our knowledge, the role of THAP1 in regulating gene expression in human neuronal cells is still unknown. In present study, using neuronal cell models for DYT6 dystonia, we found that wild-type THAP1 could regulate genes involved in network of cell growth and proliferation, while mutant THAP1 could lead to expression change of genes related to synaptic function, which include *STX3*, *STX1A*, *SOD2*, *SNCA*, *STXBP1*, and *SYT9* compared to wild-type THAP1. Synaptic function abnormalities have been reported as a pathogenic mechanism of other subtype of primary dystonia. In DYT1 dystonia, TOR1A is associated with vesicles in axons and pre-synaptic terminals (Augood et al. 2003). Mutant TOR1A leads to reduced wild-type TOR1A level and compromised the synaptic function (Granata et al. 2011). Abnormalities in synaptic vesicle recycling have also been supported by a knock-in mouse model of DYT1 dystonia (Kakazu et al. 2012). Evidence from mouse and *Drosophila* suggest that TOR1A is important for trafficking synaptic mRNAs from the nucleus to the cytosol (Speese et al. 2012; Jokhi et al. 2013). Thus, our study provides valuable insight in characterizing the pathological unfolding of primary dystonia and identifies the common pathway of different types of primary dystonia.

In present study, we also found a new transcription regulated target gene of THAP1, *SOD2*, by using ChIP-seq and luciferase report assay analysis. Protein analysis also showed the expression change of SOD2 in THAP1 overexpressing SK-N-AS cells and fibroblasts from DYT6 patients. SOD2 is exclusively located in the mitochondrial matrix and scavenges superoxide radicals. Knockout *SOD2* in mouse leads to impaired mitochondrial enzyme activity and elevated ROS (reactive oxygen species) content in synaptosomes, which finally affect the synaptic function (Flynn et al. 2011). On the contrary, overexpression of SOD2 can decrease mitochondrial superoxide and prevent Aβ-induced impairments in hippocampal synaptic plasticity in mice (Ma et al. 2011). Thus, THAP1 mutations could reduce the expression of SOD2, which might lead to synaptic dysfunction and finally causes dystonic symptom in human.

Protein stability test revealed that several mutations (C54Y, F81L) lead to decreased protein stability. Campagne and colleges (Campagne et al. 2012) reported that THAP1 mutations at positions affecting the zinc coordination, the hydrophobic core, or the C-terminal AVPTIF motif could decrease protein folding and lead to decreased protein stability. Our results experimentally supported this hypothesis. The C54Y and F81L mutation affect the zinc finger structure and AVPTIF motif, respectively, and lead to decreased protein stability. Reduced protein levels due to decreased stability might be functionally similar to other mutations reducing binding activity or transcriptional activity and responsible for dystonic phenotypes.

Our results characterized pathogenesis of different *THAP1* missense mutations and proved that although all *THAP1* mutations cause primary dystonia, the pathogenic mechanisms of mutations may be different. The limitation of current study is that this study was performed in human neuronal cell models of DYT6 dystonia rather than human iPSCs differentiated into neurons or brain tissues. However, our results could provide evidence that *THAP1* missense mutation leads to dysregulation of genes related to synaptic function, which indicates that abnormalities of synaptic function are one of the pathogeneses of DYT6 dystonia. Further studies using human neuron or human brain tissues are required in the future.

## Electronic supplementary material

ESM 1 (DOC 4250 kb)

ESM 2 (XLS 146 kb)
