# Supplementary material for: Unraveling Molecular Mechanisms of THAP1 Missense Mutations in DYT6 Dystonia
Source: J Mol Neurosci. 2020 Feb 28;70(7):999–1008. doi: 10.1007/s12031-020-01490-2 (PMC7334247; doi:10.1007/s12031-020-01490-2)
Supplement: Supplementary file 1 — (DOC 4250 kb) [file 12031_2020_1490_MOESM1_ESM.doc]

**Supplementary table 1**. List of networks generated by Ingenuity pathway analysis (wild-type THAP1 overexpression cell lines vs. empty vector transfected control cell lines) (Green: down-regulated genes; red: up-regulated genes)

| Networks | Molecules in network | Scores | Molecules | Functional description |
| --- | --- | --- | --- | --- |
| 1 | CLDN4, CYLD, DKK1, FST, IKBKE, IL32, MEST, MICA, NFKB1, NFKB1A, RARRES3, RHEBL1, RNF112, TDO2, TIFA, TNFRSF19, TNFSF18, TNFSF13B, TNIP1, TRIM21 | 26 | 20 | Cellular Growth and Proliferation, Embryonic development |
| 2 | CLN8, COL1A1, COL5A1, COL7A1, CYB5B, DACH1, HERC6, IGFBP3, KLF9, LPXN, NPAS3, PACSIN3, PARP12, PDGFD, PIP, RNF141, SEMA3B, TGFB1, TRIM24, WNT3 | 25 | 20 | Cancer, Cell to cell signaling and interaction, Connective Tissue Disorders |
| 3 | ACP2, ADCY7, ADM, APOC1, ARHGAP22, BCL6, CD36, FJX1, FTH1, GPX8, GUCY1A3, HAPLN1, NPR1, PLSCR1, PTPRO, SAP30, SOD2, STEAP1 | 25 | 18 | Infectous Disease, Lipid Metabolism, Molecular transport |
| 4 | CCL20, CEBPD, ENO3, GTF2H1, HERC5, IER3, IF16, IL11RA, MAPK12, NMU, NRG1, PTPRE, REPS2, SCLT1, SDC2, SERPINB2, STX3, TRIP6, VCL | 25 | 18 | Cell morphology, Cellular Assembly and Organization, Cellular Development |
| 5 | AGPAT3, DRAM1, FILIP1, HAPLN1, KIAA1549L, KIF26A, MPP4, PDGFRL, PLBD1, SERPINB2, SNRNP35, SRGAP2, STARD10, THSD4, TIGD1, TSPAN12, UBL3, ZNF75A | 24 | 18 | Cellular movement, Hematological System Development and Function |
| 6 | AGA, AR, CDKN1A, CELF1, DDB2, HSPA6, PTPMT1, RNF144B, SATB2, SNCA, STARD10, TRIM44, TRIM68, UBE2D3, UBE2L6, UBR3 | 23 | 17 | Post-translational modification, Organ Morphology |
| 7 | AHNAK2, ARHGAP11A, BTBD10, CDK20, CPA4, EHBP1L1, EIF1AD, GNPDA2, HCN1, MALL, MORC4, NABP1, PITPNC1, RGS17, SIPA1L2, TRIM44 | 22 | 16 | Cardiovascualr Disease, Commetive Tissue Disorders, Developmental disorder |
| 8 | C1R, CPM, DOK5, GBP4, HLA-B, IL17RD, IRF1, ISG20, PDGFC, PSMB9, SCN9A, TAP1, TAP2, TLR1, TLR3, TRIM2 | 21 | 16 | Infectious disease, Development Disorder, Hereditary Disorder |
| 9 | ANGEL2, ANXA10, ARL14EP, C11orf96, CCDC57, CMBL, ER1CH1, MXRA8, MYH15, MYO1E, NSUN5, PATL1, RGS10, SPIN4, TSPYL5, ZNF57, ZNF559 | 20 | 15 | Cancer, Neurological Disease |
| 10 | AKIP1, CNTNAP2, CRTAP, DACT1, FBXL21, FBXO3, GFOD1, MARCH11, NAT1, SAYSD1, SGK223, SKA1, TMEM65, TRMT10A, ZFR | 20 | 15 | Cell to cell signaling and interaction, Nerve System Development and Function |
| 11 | AMPD3, C1S, CCL2, DDC, FLT1, FOXP1, GEM, IGSF3, IL8, IRAK2, KCTD12, PLA2G3, SERPINB7, SESTD1, TLR2 | 20 | 15 | Cardiovascular system development and function, Cell-To-Cell Signaling and Interaction |
| 12 | ALKBH3, ARHGEF3, BHLHE41, CPE, CPEB1, CYFIP2, HBEGF, INSM1, NPL, PNMA2, PSG5, PTHLH, RGS10, SERPINB8, SLIT3 | 19 | 15 | Cellular development, Cellular Growth and Proliferation, Cell Morphology |
| 13 | ARHGAP29, CFLAR, CSRP2, GBP2, JAK1, LAPTMS, LRRC7, MAP2, MYO5A, PIK3C2G, RHOD, SPN, STK17B, SWAP70 | 18 | 14 | Cell death and survival, Cell Morphology, Cellular Assembly and Organization |
| 14 | ALDH3B1, CYB5R2, FAM69A, GLIPR1, KIAA1324, NEGR1, RAB34, RABGAP1L, RELB, SLC1A7, ST8SIA5, TM4SF19, TUBB8, USP40, XRCC4 | 18 | 14 | Cellular assembly and organization, Developmental Disorder, Endocrine System Disorders |
| 15 | AGPAT5, FYN, FZD8, GCFC2, HSD17B12, MAB21L1, NPW, OSBPL5, PDE1A, PDE9A, PHYHIPL, PPP2R3A, RERG, S100A2, TMEM25 | 17 | 14 | Cell to cell signaling and interaction, Cell signaling, Vitamin and Mineral |
| 16 | CD44, DAPK1, FOXO1, GBP1, KRT8, NCK2, PLK2, RRAD, STC1, STX1A, SYT9, THAP1, TOX, WIPF2 | 15 | 14 | Cell death and survival, Cardiovascular System Development and Function, Cellular Development |
| 17 | EMR1, FILIP1L, ICAM1, IL15, MFAP5, MICA, SNX10 | 7 | 7 | Cell-mediated immune response, Cellular Movement, Hematological System Development and Function |

**Supplementary table 2**. Common significantly dysregulated genes after stably overexpression of mutant THAP1s compared to overexpression of wild-type THAP1 in SK-N-AS cell line (With Log2 >=2)

| Gene Symbol | Gene Title | **THAP1 S21T** (log _2_) | **THAP1 F81L** (log _2_) |
| --- | --- | --- | --- |
| Genes down-regulated in mutant cell lines compared to wild type cell line | | | |
| Molecular transport and protein trafficking | | | |
| PTN | pleiotrophin | -3.17 | -2.52 |
| SNCA | synuclein, alpha (non A4 component of amyloid precursor) | -1.76 | -2.59 |
| CA12 | carbonic anhydrase XII | -1.70 | -1.41 |
| SOD2 | superoxide dismutase 2, mitochondrial | -1.40 | -1.34 |
| RRAD | Ras-related associated with diabetes | -1.25 | -1.24 |
| REPS2 | RALBP1 associated Eps domain containing 2 | -1.19 | -1.14 |
| PIP | prolactin-induced protein | -1.16 | -1.11 |
| Cellular movement and compromise | | | |
| DOK5 | docking protein 5 | -1.80 | -1.26 |
| STXBP1 | syntaxin binding protein 1 | -1.75 | -1.30 |
| ITGA7 | integrin, alpha 7 | -1.60 | -1.46 |
| DPP4 | dipeptidyl-peptidase 4 | -1.09 | -1.16 |
| Developmental and hereditary disorder | | | |
| CMBL | carboxymethylenebutenolidase homolog (Pseudomonas) | -4.43 | -2.96 |
| IGSF3 | immunoglobulin superfamily, member 3 | -2.96 | -2.35 |
| CNTNAP2 | contactin associated protein-like 2 | -3.57 | -1.58 |
| Cell morphology | | | |
| TTC39C | tetratricopeptide repeat domain 39C | -3.29 | -1.69 |
| MALL | mal, T-cell differentiation protein-like | -1.27 | -1.00 |
| Cell cycle, DNA replication | | | |
| SNX10 | sorting nexin 10 | -2.07 | -2.48 |
| RNF144B | ring finger protein 144B | -2.09 | -1.86 |
| Tissue development | | | |
| MICA | MHC class I polypeptide-related sequence A | -2.97 | -2.27 |
| ICAM1 | intercellular adhesion molecule 1 | -2.41 | -2.13 |
| FST | follistatin | -2.88 | -1.41 |
| **Genes up regulated in mutant cell lines compared to wild type cell line** | | | |
| Cellular movement and cellular compromise | | | |
| TAGLN | transgelin | 2.52 | 2.26 |
| LRRC7 | leucine rich repeat containing 7 | 2.31 | 2.09 |
| COL5A1 | collagen, type V, alpha 1 | 1.34 | 1.23 |
| Developmental disorder, hereditary disorder | | | |
| AGPAT3 | 1-acylglycerol-3-phosphate O-acyltransferase 3 | 2.22 | 2.44 |
| Cell morphology | | | |
| PDE1A | phosphodiesterase 1A, calmodulin-dependent | 3.76 | 1.79 |
| Cell cycle, DNA replication | | | |
| MCPH1 | microcephalin 1 | 1.10 | 1.06 |
| Tissue development | | | |
| BOK | BCL2-related ovarian killer | 1.54 | 1.36 |


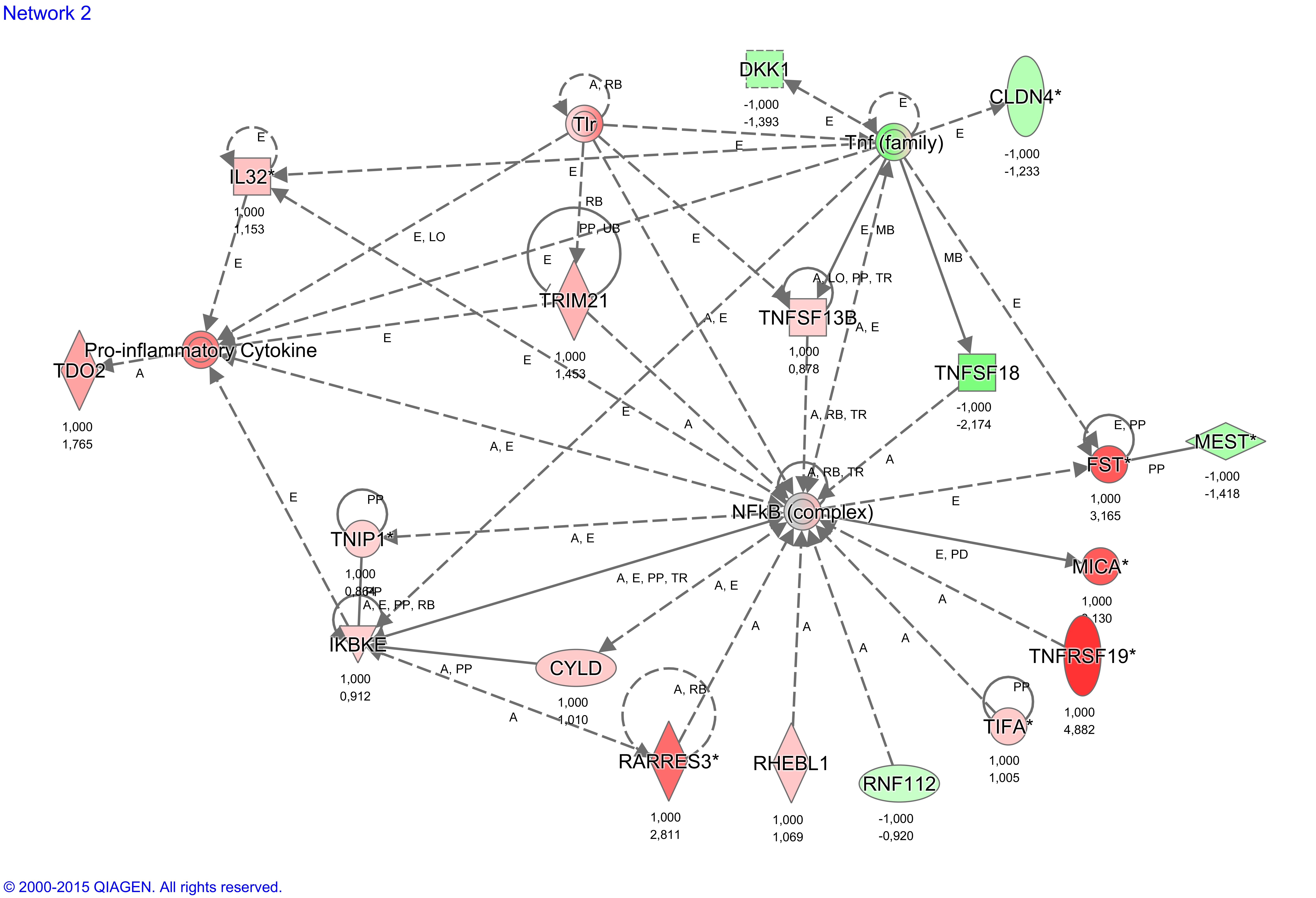
**Supplementary figure 1**. The significant molecular and cellular function change in THAP1 wild type cell line compared to empty vector cell line generated by Ingenuity pathway analysis. Cell growth and proliferation network was presented. (A, activation / deactivation; E, expression; LO, localization; MB, biochemical modification; PD, protein-DNA binding; PP, protein-protein binding; RB, regulation of binding; TR, transcription.) (Green: down-regulated genes; red: up-regulated genes)


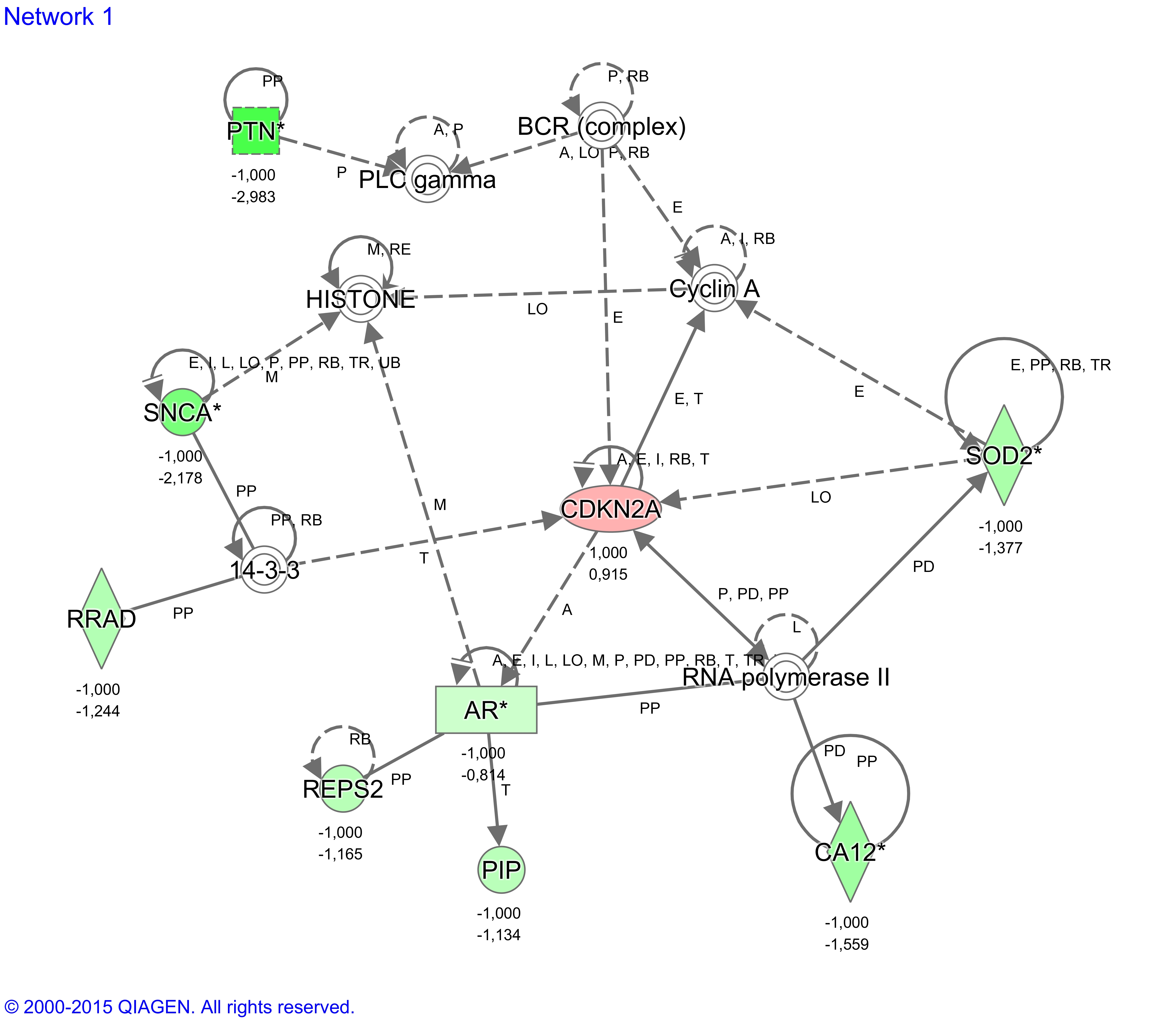


**Supplementary figure 2**. The significant molecular and cellular function change in THAP1 mutant cell lines compared to THAP1 wild-type cell line generated by Ingenuity pathway analysis. Molecular transport and protein trafficking network was presented. (A, activation / deactivation; E, expression; LO, localization; MB, biochemical modification; PD, protein-DNA binding; PP, protein-protein binding; RB, regulation of binding; TR, transcription.) (Green: down-regulated genes; red: up-regulated genes)
